# Clinical indications and protocol considerations for selecting initial body weight support levels in gait rehabilitation: a systematic review

**DOI:** 10.1186/s12984-024-01389-8

**Published:** 2024-06-07

**Authors:** Sanne Ettema, Geertje H. Pennink, Tom J.W. Buurke, Sina David, Coen A.M. van Bennekom, Han Houdijk

**Affiliations:** 1 Research and Development, Heliomare Rehabilitation, Wijk aan Zee, the Netherlands; 2 Department of Human Movement Sciences, University of Groningen, University Medical Center Groningen, Groningen, the Netherlands; 3 Department of Human Movement Sciences, Vrije Universiteit Amsterdam, Amsterdam Movement Sciences, Amsterdam, the Netherlands; 4 Department of Movement Sciences, KU Leuven, Leuven, Belgium; 5 Department of Public and Occupational Health, Amsterdam UMC, Amsterdam, the Netherlands

**Keywords:** Body weight support, Rehabilitation, Gait training

## Abstract

**Background:**

Body weight support (BWS) training devices are frequently used to improve gait in individuals with neurological impairments, but guidance in selecting an appropriate level of BWS is limited. Here, we aim to describe the initial BWS levels used during gait training, the rationale for this selection and the clinical goals aligned with BWS training for different diagnoses.

**Method:**

A systematic literature search was conducted in PubMed, Embase and Web of Science, including terms related to the population (individuals with neurological disorders), intervention (BWS training) and outcome (gait). Information on patient characteristics, type of BWS device, BWS level and training goals was extracted from the included articles.

**Results:**

Thirty-three articles were included, which described outcomes using frame-based (stationary or mobile) and unidirectional ceiling-mounted devices on four diagnoses (multiple sclerosis (MS), spinal cord injury (SCI), stroke, traumatic brain injury (TBI)). The BWS levels were highest for individuals with MS (median: 75%, IQR: 6%), followed by SCI (median: 40%, IQR: 35%), stroke (median: 30%, IQR: 4.75%) and TBI (median: 15%, IQR: 0%). The included studies reported eleven different training goals. Reported BWS levels ranged between 30 and 75% for most of the training goals, without a clear relationship between BWS level, diagnosis, training goal and rationale for BWS selection. Training goals were achieved in all included studies.

**Conclusion:**

Initial BWS levels differ considerably between studies included in this review. The underlying rationale for these differences was not clearly motivated in the included studies. Variation in study designs and populations does not allow to draw a conclusion on the effectiveness of BWS levels. Hence, it remains difficult to formulate guidelines on optimal BWS settings for different diagnoses, BWS devices and training goals. Further efforts are required to establish clinical guidelines and to experimentally investigate which initial BWS levels are optimal for specific diagnoses and training goals.

**Supplementary Information:**

The online version contains supplementary material available at 10.1186/s12984-024-01389-8.

## Background

Over the last decades, gait rehabilitation technology has seized a firm spot in the rehabilitation of individuals with neurological gait disorders, such as stroke, spinal cord injury, cerebral palsy and multiple sclerosis [1, 2]. Rehabilitation technology is widely used to assess gait quality and behavior [3], and to improve gait function through the use of supportive training devices [1]. Many of these training devices have found their way into clinical practice and have been implemented within rehabilitation centers. Amongst these rapid innovative developments, there has been great interest in body weight support (BWS) devices. These devices have emerged as an appealing option to clinicians as they stimulate early gait training and reduce the physical burden on a therapist [4].

The use of BWS devices has shown promise in improving walking ability and avoiding the development of malfunctional compensatory movement patterns in various patient groups [4–6]. Generally, BWS is provided by an overhead suspension mechanism and a harness that apply vertical forces on a person’s pelvis or trunk causing partial weight reduction [7]. Initially, BWS training was mainly offered to individuals with a spinal cord injury, as its working mechanism was primarily associated with neuroplasticity [8, 9] and functional re-organization of neuronal networks [10]. Then, BWS devices were also used for other diagnoses, as they reduce the load on the lower limbs [11], improve vertical alignment and trunk stability [12], enhance gait initiation [13] and improve physical fitness [14]. It is also thought that BWS reduces the fear of falling through prevention mechanisms that ensure a safe walking environment [4].

Recently, BWS devices have developed from stationary, treadmill-coupled devices to more elaborate mobile and ceiling-mounted systems with multiple degrees of freedom that can be used during overground walking [15]. The current developments in BWS devices accompany the trend towards promoting active participation in training and providing assist-as-needed based on patient-specific requirements [16]. Roughly, four main categories of BWS devices can be distinguished: frame-based constructions (either stationary or mobile) and ceiling mounted devices (either unidirectional or multidirectional). Well-known examples of frame-based constructions are the Woodway Loko system (stationary, Woodway USA Inc., USA) and the LiteGait (mobile, Mobility Research, USA), whereas examples of ceiling-mounted devices are the ZeroG (unidirectional, Aretech, USA) and the RYSEN (multidirectional, Motek Medical, The Netherlands).

Although all different types of BWS devices are frequently used in rehabilitation programs, guidance in selecting an appropriate support level is limited. In literature, providing BWS up to 30% is generally recommended as this is shown to allow walking with close to normal kinematics [17, 18]. However, gait rehabilitation depends on more factors than solely normal gait kinematics and therapists may consider different reasons to select BWS levels, such as patient-specific characteristics or training goals. Guidelines on clinically relevant and feasible BWS selection are currently lacking and therapists often subjectively determine BWS levels based on visual inspection and patient’s feedback.

This systematic review aims to describe the initial BWS levels used during gait training, the rationale for this selection, the clinical goals that are aligned with the use of BWS and whether these differ between diagnoses. Moreover, the study aims to describe whether pursued training goals are more likely to be achieved at particular BWS levels and within a particular diagnosis. Insights from this study can serve as a first step towards developing clinical guidelines.

## Methods

The selection process of identification, screening, eligibility and inclusion was performed in accordance to the Preferred Reporting Items for Systematic Reviews and Meta-Analyses (PRISMA) guidelines for reviews (Appendix 1). Prior to the search, this review was registered in PROSPERO (international prospective register of systematic reviews; registration number CRD42022367172).

### Search strategy

Three electronic databases (PubMed, Embase, Web of Science) were assessed and searched on April 20th 2023. The search strategy was developed with the help of a medical librarian and refined in group discussion after preliminary searches. The final search strategy (Appendix 2) included a list of terms relating to or describing the population (individuals with neurological disorders), intervention (BWS training) and outcome (gait).

### Study selection and inclusion criteria

After exclusion of duplicate articles, two independent researchers (SE and GP) selected eligibility based on title and abstract. Thereafter, the researchers assessed potentially relevant articles by reading full-text. Any in- and exclusion conflict between the researchers was discussed to reach consensus. In case of persistent disagreement, a third independent reviewer (TB) could be consulted. Of the studies included in the review, reference lists were screened for other relevant studies that may have been missed in the search. The following inclusion criteria were used when selecting articles: [1] the population consisted of adults (> 18 years of age) with neurological disorders (i.e. traumatic brain injury (TBI), stroke, multiple sclerosis (MS), cerebral palsy (CP) or spinal cord injury (SCI)); [2] the only intervention used was a BWS device (i.e. no exoskeletons and not combined with virtual reality, electrostimulation, conventional gait training, etc.); [3] the BWS device was used during forward walking; [4] the study design included a clinical protocol that investigated the effectiveness of a BWS training intervention; [5] at least two participants were included; [6] the BWS level was reported; [7] the article was written in English and was not a conference abstract, review, letter to the editor or protocol.

### Data extraction, outcome measures and risk of bias assessment

The following information was extracted from the included articles: [1] participant characteristics (i.e. sample size, sex, age, time since injury, mobility level); [2] device characteristics (i.e. walking surface, type of BWS device); [3] BWS characteristics (i.e. the BWS level and rationale for selecting the BWS level); [4] training characteristics (i.e. duration of training period/sessions and frequency); [5] training goals reflected by the outcome measures; [6] attainment of training goals.

The rationale for the selected BWS level in the included studies was categorized into ‘rationale provided’ and ‘rationale not provided’. For the studies that provided a rationale, it was determined whether it was based on qualitative or quantitative criteria.

Training goals were categorized into pre-defined categories (according to the reported outcome measures) by two independent researchers (SE and GP; Appendix 3). The categories were defined based on chapters of the International Classification of Functioning (ICF) framework. When more than one outcome measure was reported, articles could be allocated into multiple categories.

When BWS levels were variable over a training period, the level that was applied at the start of the training period was used for further analysis. When only individual BWS values were reported, the average value over all participants was used for further analysis. If only a range of optional values was reported, we picked the middle of the range for further analysis.

Two researchers (SE and GP) independently assessed the risk of bias of the included articles using the Newcastle-Ottawa Scale (NOS [19]). The NOS was slightly modified for the purpose of the current review and contained items on participant selection and outcome assessment (Appendix 4) to check if these items were properly reported. The risk of bias assessment did not assess the design of the included studies. For each of the six items included, studies could be awarded a maximum of one star. Total NOS scores range from 0 to 6, with lower risk of bias reflected by higher scores.

## Results

### Literature search

The search identified a total of 3004 articles. Removing duplicates resulted in 1879 articles to be screened based on title and abstract. Consequently, 226 articles were identified as possibly relevant. The majority of the articles were excluded after full text screening, because a BWS device was not the only intervention used (*n* = 79), the article was a conference abstract (*n* = 47), there was no training intervention (*n* = 26), BWS characteristics were not reported (*n* = 20), there was limb assistance during stepping (*n* = 8), other diagnoses were involved (*n* = 7), the article was a single case-study (*n* = 4) or the population was already reported in another included study (*n* = 3). Reference list screening resulted in one additional article. Eventually, 33 studies were included in this review (Appendix 5).

### Study characteristics

In total, the included studies reported the outcomes on 156 persons with a SCI, 204 persons after stroke, 22 persons with TBI and 11 persons with MS (Table 1). No studies on CP were found that matched the inclusion criteria. In general, the study sample size, sex, age, diagnosis and time since injury were well reported. Considerable heterogeneity was noted in terms of the participants’ characteristics such as sex (67% male), age (range: 18–93 years), time since injury (range: 524 days-13 years) and the level of mobility (walking with or without assistance versus wheelchair dependent).

**Table 1:** Summary of all study characteristics

	Participant characteristics					Device characteristics			BWS characteristics			Training characteristics			Outcomes	
Author, year	Group M: F	Diagnosis	Age [y]	TSI	Mobility	Surface	Device type	Device name	BWS [%]	Type of support (fixed/variable) during training period	Reason for (changing) % BWS	Duration of the training period	Frequency of training [days/wk]	Training duration [min]	Outcome measures / training goals	Goals achieved
Abel, 2002 [38]	3:4	SCI	38 (18–60)	-	Wheelchair dependent	T	FB-stationary	custom-made	25	Variable	-	9 w	5	-	Mobility of joint functions	Increased walking speed and duration
Alexeeva, 2011 [22]	12:2	SCI	36 (13)	8 (10) y	Wheelchair dependent	O	CM-single rail	Maine AntiGravity Systems	30	Fixed	-	13 w	3	-	Gait pattern functions, muscle power functions, mental functions, involuntary movement reaction functions	Improved walking speed, muscle strength and well-being
Alexeeva, 2011 [22]	8:1	SCI	43 (16)	5 (4) y	Wheelchair dependent	T	CM-single rail	Maine AntiGravity Systems	30	Fixed	-	13 w	3	-	Gait pattern functions, muscle power functions, mental functions, involuntary movement reaction functions	Improved walking speed, muscle strength and well-being
Effing, 2006 [39]	3:0	SCI	48 (3)	96 (90) m	Transfer without help and walk with walking aids	T	FB-stationary	Woodway Loco-system	30	Variable	-	12 w	5	30	Mental functions	Increased in quality of life, activities of daily living and walking performance
Gazzani, 1999 [40]	5:2	SCI (6) TBI (1)	48 (18)	150 (236) m	Wheelchair dependent	T	FB-mobile	WARD	39	Variable	Maintain balance without risk	1–2 m	3	30	Functional ability, gait pattern functions, metabolic functions, cardiovascular functions	Clinical scores improved,, speed increased, energy cost and heart rate improved
Giangregorio, 2006 [24]	11:2	SCI	29 (8)	8 (7) y	-	T	FB-stationary	Woodway Loco-system	68	Variable	Trunk and limb alignment, prevent dangling	12 m	3	-	Neuromusculoskeletal functions	Increased body lean mass, muscle cross-sectional area and whole-body bone density
Gorassini, 2009 [41]	16:3	SCI	46 (18)	4.8 (7.4) y	-	T	FB-stationary	Custom-made	50	Variable	-	14	5	60	Muscle power functions	Increased muscle activity, decreased duration of muscle activity
Hicks, 2005 [42]	11:2	SCI	29	7.4 (7) y	Wheelchair dependent	T	FB-stationary	Woodway Loco-system	73	Variable	Trunk and limb alignment	48	3	15–45	Gait pattern functions, mental functions	Improved walking ability, walking speed, walking distance and well-being
Ivanenko, 2003 [43]	*n* = 11	SCI	45 (16)	-	-	T	FB-stationary	Custom-made	75	Variable	Patient’s improvement	4–12			Mobility of joint functions, muscle power functions	Timing of muscle activation became comparable to healthy controls and temporal structures were similar
Martin Ginis, 2007 [44]	11:3	SCI	28.8 (8)	7.4 (6.9) y	-	T	FB-stationary	Woodway Loco-system	60	Variable	Trunk and limb alignment, prevent dangling	12 m			Sensory functions and pain	Decreased pain
Musselman, 2009 [45]	2:2	SCI	43.5 (15.3)	7.3 (10.6) y	Wheelchair dependent, using assistive devices	T	FB-stationary	Custom-made	27	Variable	-	3 mo	5	60	Functional ability, gait pattern functions	Improved walking speed, walking endurance, obstacle clearance and stair climbing
Phillips, 2004 [46]	8:1	SCI	-	8 (3) y	Wheelchair dependent	T	FB-stationary	Woodway Loco-system	65	Variable	-	6 m	3	-	Metabolic functions	Increased muscle glycogen and improved blood glucose regulation
Piira, 2020 [23]	13:8	SCI	18–70	> 2 y	Wheelchair dependent	T	FB-stationary	Vigor equipment	33	Variable	-	6 m	3–5		Functional ability, mental functions, gait pattern functions	Quality of life did not improve
Protas, 2001 [47]	3:0	SCI	43 (8)	9 (6) y	Wheelchair dependent	T	FB-stationary	STAT device	40	Variable	-	12 w	5	60	Gait pattern functions, metabolic functions, muscle power functions, control of voluntary movement functions	Increased walking speed, walking endurance and reduced oxygen cost
Stewart, 2004 [48]	8:1	SCI	31 (3)	8.1 (2.5) y	Wheelchair dependent	T	FB-stationary	Woodway Loko-system	65	Variable	Patient’s improvement	60 m	3		Cardiovascular functions, muscle power functions	Improved walking speed, walking endurance, increased muscle cross-sectional area and fibre size
Chua, 2020 [49]	7:4	Stroke	53 (22)	524 (811) d	FAC 2–4	T	FB-stationary	VASST II	17	Variable	Clinically determined	5 w	-	-	Functional ability	Improvement in 6MWT and BBS scores
Combs, 2013 [20]	4:11	Stroke	60 (11)	4 (3) y	Ambulatory with or without use of assistive devices	T	FB-mobile	Lite Gait	30	Variable	Speed	8 w	-	20	Gait pattern functions	Improvement in relative phase in the direction of healthy walking
Combs, 2014 [25]	4:6	Stroke	56.2 (8)	62.3 (48.6) m	Ambulatory with or without use of assistive devices	T	FB-mobile	Lite Gait	30	Variable	Speed of 2mph was achieved	2 w	5	30	Gait pattern functions	Improved walking speed,
Gama, 2017 [36]	7:7	Stroke	59 (8)	60 (56) m	Ambulatory with or without use of assistive devices	T	CM-single rail	Custom-made	30	Variable	Trunk and limb alignment, limb loading	6 w	3	45	Gait patterns functions	Improved walking speed, scores on 6MWT and independence
Gama, 2017 [36]	6:8	Stroke	58 (10)	54 (42) m	Ambulatory with or without use of assistive devices	O	CM-single rail	Custom-made	30	Variable	Trunk and limb alignment, limb loading	6 w	3	45	Gait patterns functions	Improved walking speed, scores on 6MWT and independence
Geroin, 2011 [35]	6:4	Stroke	63.3 (6)	26.7 (5.1) m	Ability to walk independently for at least 15 m with or without walking aids	T	P	Gait Trainer	30	Variable	-	2	5	50	Gait pattern functions, functional ability	Improvement in 6MWT and 10MWT
Graham, 2018 [21]	7:8	Stroke	60 (13)	48 (65) m	Ambulatory with or without use of assistive devices	T	FB-stationary	KineAssist	30	Variable	Speed > 0.08 m/s faster than 0% BWS	6 w	3	30	Gait pattern functions, functional ability	Improved walking speed
Kim, 2020 [50]	12:2	Stroke	55 (12)	6 (3) m	-	T	FB-stationary	Custum-made	30	Variable	-	4 w	5	30	Gait pattern functions, functional ability	Improvement in Fugl-Meyer score, Time Up and Go test, 10MWT
Kim, 2014 [51]	8:4	Stroke	53 (9)	11.8 (3.5) m	FAC 3	T	FB-stationary	Custom-made	30	Variable	-	3 w	6	30	Gait pattern functions	Step length, walking ability and stance phase improved
Miller, 2002 [52]	0:2	Stroke	87 93	10 3 y	-	T / O	FB-mobile	LiteGait	40	Variable	Heel ground contact	6–7 w	2–3	-	Gait pattern functions, functional ability	Improvements in 10MWT, BBS, step length and 10MWT
Moore, 2010 [53]	14:6	Stroke	50 (15)	13 (8) m	Walk with assistive device	T	FB-stationary	Custom-made	40	Variable	Speed < 0.2 m/s than overground	4 w	2–5		Gait pattern functions, functional ability, cardiovascular functions	Increase in stepping practice
Ribeiro, 2013 [26]	*n* = 11	Stroke	56.5 (8.3)	33.4 (25) m	FAC 3–5	T	FB-stationary	Gait Trainer	30	Variable	Exercise tolerance	4 w	3	30	Gait pattern functions, functional ability, mobility of joint functions	Improved motor function, symmetry, walking speed, stride length
Sousa, 2011 [54]	8:4	Stroke	53 (8)	5 (3) y	Ability to walk 10 m with or without assistive devices	O	CM-single rail	Custom-made	30	Fixed	Increase muscle activation and intensity	6 w	3	45	Mobility of joint functions, gait pattern functions	Improved walking speed, symmetry, stride length, clearance and limb rotation
Sullivan, 2002 [55]	7:1	Stroke	64 (13)	27 (14) m	Ability to walk 10 m with or without assistive devices	T	FB-stationary	Custom-made	33	Variable	Increase exercise tolerance and maintain limb kinematics	4 w	3	20	Gait pattern functions	Improved walking speed
Takao, 2015 [34]	8:2	Stroke	59 (13)	35 (33) m	-	T	FB-mobile	Biodex Unweighting system	20	Fixed	-	4 w	3	-	Gait pattern functions, functional ability	Improved walking speed
Trueblood, 2001 [56]	*n* = 13	Stroke	62.5 (11)	10.9 (8.6) m	Walk with assistive devices	T	FB-mobile	Lite Gait	40	Variable	Trunk and limb alignment, symmetrical weight bearing, allows for heel strike	6 w	3	-	Gait pattern functions, muscle power functions	Improvement in walking speed, step length and limb support
	*n* = 13	Stroke	61.7 (11)	9.8 (5.6) m	Walk with assistive devices	T	FB-mobile	Lite Gait	40	Variable	Trunk and limb alignment, symmetrical weight bearing, allows for heel strike	8 w	-	-	Gait pattern functions, muscle power functions	Improvement in double support
Esquenazi, 2013 [57]	4:4	TBI	41.9 (16.8)	150.4 (111.6) m	Walk with canes, crutches, walkers or wheelchair dependent	T	FB-mobile	Lite Gait	15	Variable	-	6 w	3	45	Gait pattern functions, functional ability	Increased walking speed, symmetry and walking distance
Esquenazi, 2017 [27]	6:1	TBI	38 (11)	> 12 m	Ability to walk 10 m with or without assistive devices	T	FB-stationary	G-EO	15	Fixed	-	6–8 w	3	45	Gait pattern functions, functional ability	Increased walking speed
Esquenazi, 2017 [27]	3:4	TBI	44 (17)	> 12 m	Ability to walk 10 m with or without assistive devices	T	FB-mobile	LiteGait	15	Fixed	-	6–8 w	3	45	Gait pattern functions, functional ability	Increased walking speed
Pilutti, 2011 [58]	2:4	MS	48 (9)	12 (7) y	-	T	FB-stationary	Woodway Loco-system	78	Variable	Trunk and limb alignment, prevent dangling	12 w	3	-	Mental functions, cardiovascular functions	Increased walking speed, increased training intensity and reduction of fatigue
Pilutti, 2016 [59]	3:2	MS	48 (4)	13 (11) y	-	T	FB-stationary	Woodway Loco-system	72	Variable	Intensity	12 w	-	30	Mental functions, cardiovascular functions	Improved quality of life, reduced fatigue

Participant characteristics, device characteristics, BWS characteristics, training characteristics and outcome measures. Abbreviations: M:F: male: female; TSI: time since injury; BWS: body weight support; SCI: spinal cord injury; TBI: traumatic brain injury; MS: multiple sclerosis; T: treadmill; O: overground; FB: frame-based; CM: ceiling-mounted; -: information not available

Persons with a SCI were included in 14 studies, persons after stroke in 15 studies, persons with MS and persons with TBI both in two studies. Concerning the different categories of BWS devices, 22 studies used frame-based stationary devices, eight studies frame-based mobile devices and three studies ceiling-mounted unidirectional devices. There was no data available for ceiling-mounted multidirectional devices. In 30 studies, BWS training was performed on a treadmill, whereas overground training was performed in four studies. In total, 11 different types of BWS devices were used. Custom-made devices were most often used (*n* = 12), followed by the Woodway LOKO system (*n* = 8, Woodway USA Inc., USA) and the LiteGait (*n* = 6, Mobility Research, USA). Other types of BWS devices were used in two studies or less.

**Fig. 1:**
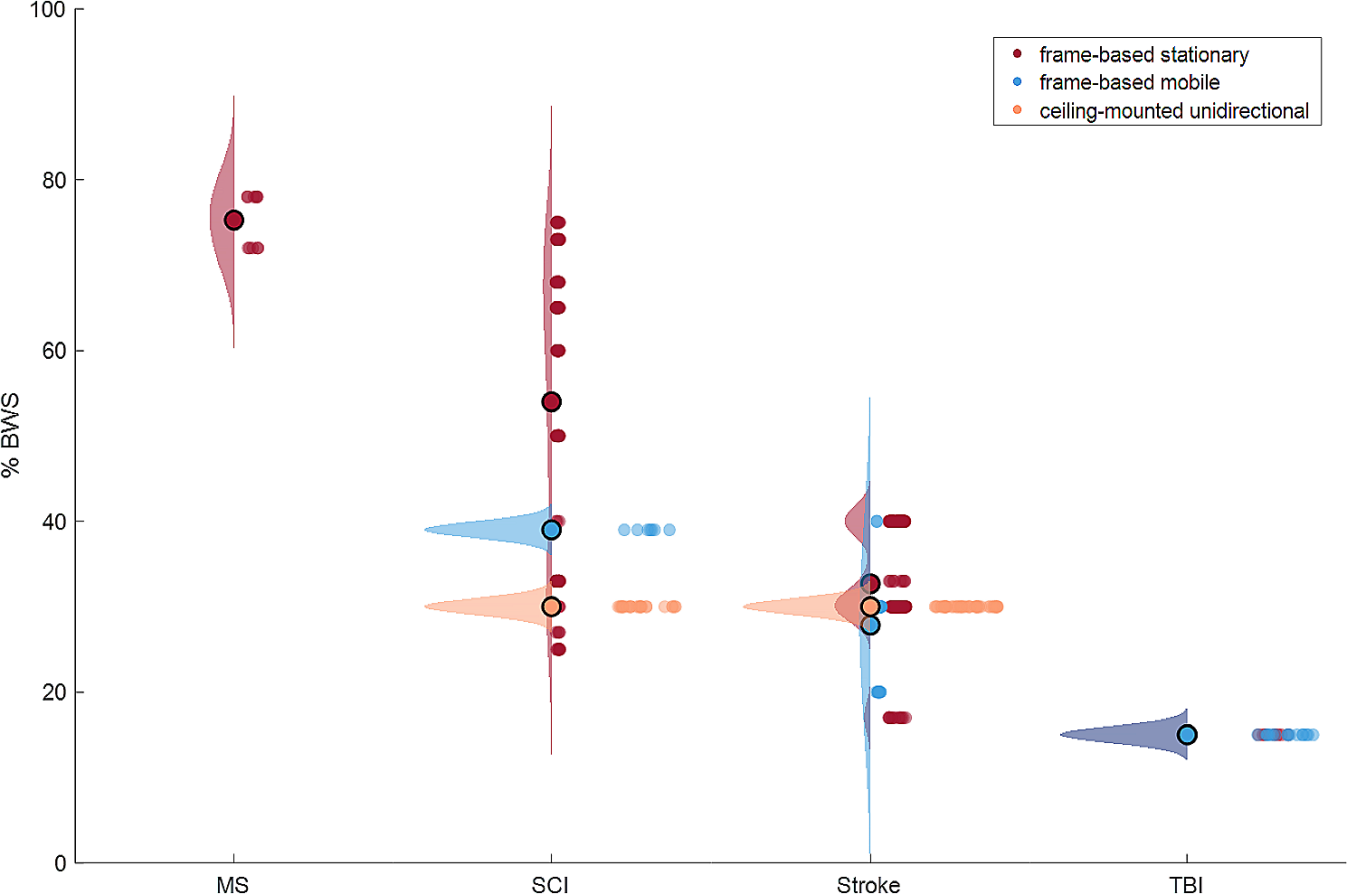
Vertical raincloud plot of the Body Weight Support (BWS) levels used for different diagnoses (x-axis) and types of BWS devices (in different colors). The left half of the raincloud plot shows the group distribution and group mean (large open dots) for each diagnosis and BWS device based on the number of participants that was included in the corresponding studies. The right half of each raincloud plot shows the data for individual studies (small dots). MS: multiple sclerosis; SCI: spinal cord injury; TBI: traumatic brain injury

The BWS levels in the included studies ranged from 17 to 78% between all included studies (median: 30%, interquartile range: 12.5%). The BWS levels were highest for individuals with MS (median: 75%, interquartile range: 6%), followed by individuals with a SCI (median: 40%, interquartile range: 35.0%), individuals after stroke (median: 30%, interquartile range: 4.75%) and individuals with TBI (median: 15%, interquartile range: 0%, Fig. 1). In 31 studies, the BWS level was variable over the training period and was adapted to the capabilities of the patients. A fixed BWS level was used in two studies. Variable BWS levels were reported differently between studies. 14 studies reported only the BWS level applied at the start of the training period, mentioning maximum (*n* = 1), average (*n* = 2), optional (*n* = 3) or fixed (*n* = 8) values. Seven studies reported average BWS levels at the start and end of the training period and one study reported these start and end levels per individual. BWS level progression for multiple time points was reported by nine studies, mentioning average (*n* = 5) and individual (*n* = 4) levels.

**Fig. 2:**
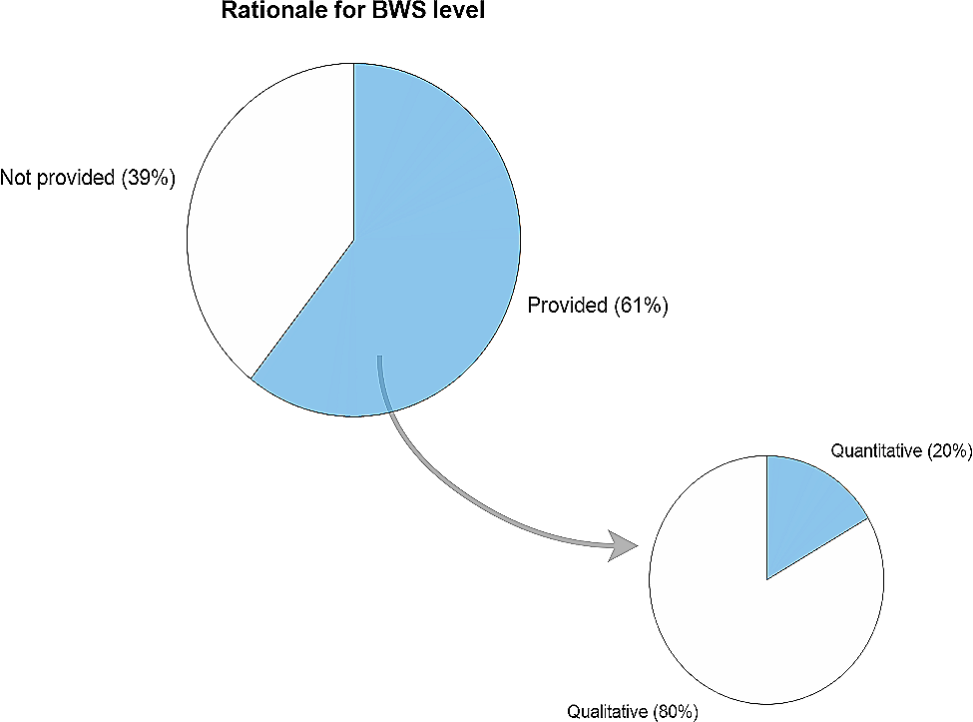
Rationale for selecting Body Weight Support (BWS) levels

In 13 studies, a rationale for selecting the level of BWS was not provided. Among the provided reasons in the other 20 studies, 16 studies provided qualitative descriptions for the applied BWS level, e.g. “the level of BWS was progressively decreased based on speed and quality demand” [20], whereas four studies used quantitative reasons to select the level of BWS, e.g. “we selected the BWS level that resulted in walking > 0.08 m/s faster than 0% BWS” [21] (Fig. 2).

**Fig. 3:**
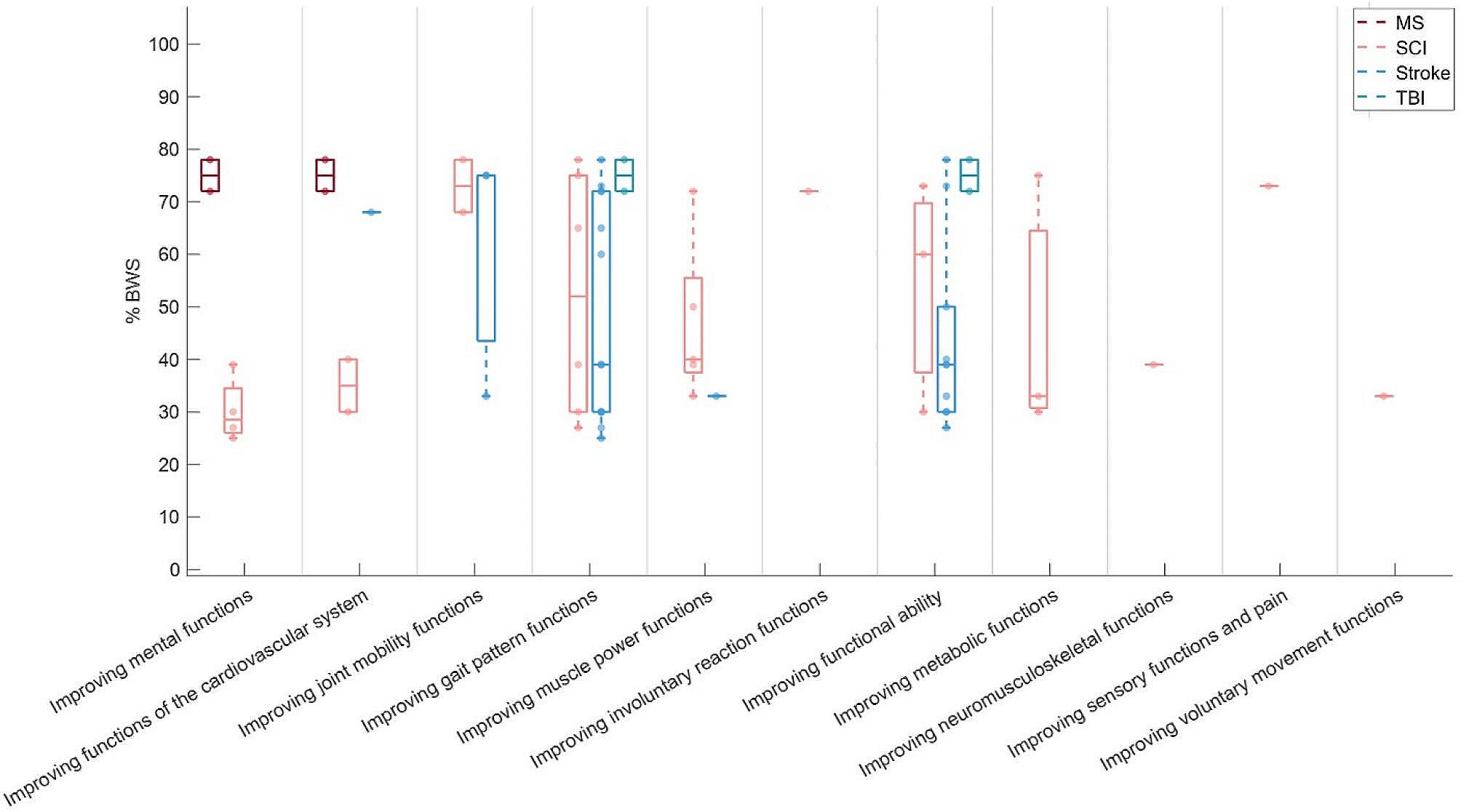
Boxplots of the Body Weight Support (BWS) levels used for different training goals. The boxplots show the following: box, interquartile range (IQR; 25th -75th centiles); upper whisker, upper adjacent; lower whisker, lower adjacent; circle, median. MS: multiple sclerosis; SCI: spinal cord injury; TBI: traumatic brain injury

The included studies reported a wide variety in training goals as reflected by the studies’ outcome measures (Fig. 3). The majority of the studies (*n* = 22) had improving gait pattern functions as a rehabilitation goal, which included outcome measures such as speed, 10 m Walk Test, step length, step width and gait symmetry (Appendix 3). In 14 studies, improving functional ability was set as rehabilitation goal. Reported outcome measures included scores on functional and clinical tests, including the Berg Balance Scale, Functional Ambulation Category, Motricity Index and Fugl-Meyer Assessment.

For individuals with a SCI, all 11 different training goals were pursued by BWS training over all included studies. For individuals after stroke, five goals were reported (improving functions of the cardiovascular system, joint mobility functions, gait pattern functions, muscle power functions and functional ability). For individuals with MS, two different training goals were pursued over all included studies (improving mental functions and functions of the cardiovascular system). For individuals with TBI, also two training goals were reported (improving gait pattern functions and functional ability). For most of the training goals, the BWS level varied between 30 and 75%.

The rationales provided for BWS level selection differed between diagnoses and between studies that had the same training goals. For individuals with a SCI, the BWS level was often selected to ensure an upright trunk and prevent buckling of the knees. For individuals after stroke, the BWS level was often selected based on walking speed. The rationale for BWS level selection also differed between studies that had the same training goals. For instance, BWS levels for the goal of improving functions of the cardiovascular system were based on the alignment of the trunk and limbs, training intensity, individual improvement and walking speed. For the goal of improving gait pattern functions, selection was frequently based on walking speed, but also on the alignment of the trunk and limbs and gait kinematics.

All included studies achieved most of their training goals. For all diagnoses, BWS gait training resulted in increased walking speed after several weeks of training, with applied BWS levels ranging from 20 to 78%. For individuals with a SCI, BWS gait training was frequently beneficial for improving walking endurance. In three studies on individuals with a SCI, training goals were not attained, i.e. balance control [22] and quality of life [23] did not improve when using 30% BWS and bone density did not improve when using 68% BWS [24]. For individuals after stroke, BWS gait training often improved step length and step symmetry. However, in two studies, increases in step length and step symmetry were not found when using 30% BWS [25, 26]. For individuals with TBI, gait training did not improve swing and stance time [27], when using 20% BWS.

### Risk of bias assessment

The mean NOS score and standard deviation were 4.1 ± 1.2 (range: 2–6). For most studies, stars were awarded for descriptions of training duration and frequency, the BWS level and participant characteristics (Appendix 6). Overall, stars were more often withheld for items related to participants screening, selection and follow-up of study groups. For these items, information was often not explicitly described.

## Discussion

This systematic review provided a detailed overview of initial BWS levels used during clinical gait training, and showed that BWS levels differ considerably between studies included in this review and tend to differ between diagnoses, types of BWS devices and within training goals. Our findings show that consensus on selecting BWS levels is currently lacking, as well as clarity on the underlying reasons for selecting a BWS level. The results of this review seem to reflect the uncertainty within clinical practice about what BWS levels should be used. Findings from this review can serve as a starting point for the development of guidelines that can be used in rehabilitation programs.

In total, we identified 33 studies that applied BWS training to improve gait in individuals with neurological impairments. These studies were, however, not equally distributed over the included diagnoses and BWS device types. Specifically, data for individuals with MS and TBI were underrepresented in literature and no studies on adults with CP were included. Moreover, the majority of the included studies used frame-based stationary devices, whereas none of them used multidirectional ceiling-mounted devices. It should be acknowledged that the limited and heterogeneous data available for specific diagnoses and devices hamper intercomparisons. However, our results clearly show that variety exists in the amount of BWS applied during gait training, with values ranging from 17 to 78% over all included studies.

In 39% of the studies included in this review, a rationale for selecting the BWS level was not reported. Although the majority of the included studies did provide a rationale for selected BWS levels, the arguments provided lack clarity to account for the variation in the BWS levels found, as the rationale provided was based on qualitative criteria in 80% of the cases. The variety in BWS levels and the high number of qualitative rationales provided could reflect the current practice in which the BWS level is often determined subjectively [18]. Potentially, the wide range of BWS levels could also be due to differences in specific rehabilitation goals of the studies, but our results show that even within most of the training goals the range of applied BWS levels is substantial. The wide range of BWS levels corresponds with the diversity in rationales provided for BWS level selection within the training goals. This diversity could be explained by the large number of different rationales provided in general, but also by the fact that most of the goals were pursued by multiple diagnoses, whereas the rationales seemed to be slightly dependent on diagnosis. However, this does not necessarily mean that the strategy for BWS level selection differs between diagnoses, as researchers from the same field may have adopted research protocols from other research groups. Due to the limited number of rationales extracted from the included studies and the diversity of rationales within training goals, comparing the strategies for BWS level selection between training goals was not possible in this study. Future research may determine how BWS levels should be tailored to specific training goals [28].

Our results may argue for a patient and training-tailored selection of BWS levels within future guidelines. Despite the fact that data is limited for some of the included diagnoses and BWS devices, our results indicate that slightly higher BWS levels seem to be used for individuals with MS and SCI than for individuals after stroke and TBI. However, differences between diagnoses need to be interpreted with caution, as they likely result from potential confounders, such as differences in patient and training characteristics. Factors such as severity of the disorder, age, time since injury, cognitive level, training goals, within therapy changes of the BWS level and frequency and duration of training may have had a larger influence on BWS level selection than the diagnosis itself. In the studies included in this review, individuals with a SCI were often wheelchair dependent, whereas individuals after stroke were able to walk with assistive devices. Consequently, differences in mobility level between diagnoses could explain why the applied BWS levels were slightly higher for individuals with a SCI compared to individuals after stroke. The possibility to change BWS levels within the training period could be considered another confounder. Studies that allowed to change BWS levels during the training period may have used higher start levels than studies that used a fixed level over the whole training period. Since movement strategies can already be affected by small adaptations in tasks [29], it seems to be important to accurately tune the level of BWS to specific circumstances, taking into account the potential confounders described above [30]. This approach is in line with the assist-as-needed principle, indicating that the amount of support is based on individual requirements [16], and can be a strategy to determine BWS levels in future guidelines.

Only a limited set of cross-sectional studies (that were not included in this review) systematically investigated effects of different BWS levels on outcome measures such as spatiotemporal gait parameters, muscle activity and metabolic costs [4, 12, 13, 31, 32]. From these studies it is known that higher BWS levels reduce metabolic costs [32], as higher BWS levels require less muscle activity [33]. Moreover, increased BWS levels seem to reduce step length [13] and increase step width [12]. However, in most of these cross-sectional studies, only a few BWS levels have been applied and therefore it is unclear how gait related parameters change over a full range of BWS levels. In their systematic review, Apte et al. [18] pooled together multiple cross-sectional studies to predict changes in gait over a full range of BWS levels. Despite that their results provide insight in how gait could change by increasing levels of BWS, they may be distorted by the influence of different diagnoses and BWS devices as shown in this systematic review.

Our results show that all included studies attained most of their training goals, regardless of the applied BWS level. It should be noted that the majority of the studies in this review (*n* = 28), did not include a control group without BWS. In the absence of a control group without BWS, the added value of BWS in comparison to conventional gait training remains unknown. Results from five studies that included control groups lack clarity on the effectiveness of BWS gait training in general and the applied BWS level specifically. Two of the included studies [34, 35] found greater improvements in gait speed during BWS training, whereas three other studies showed greater improvements in speed [25], balance control [22], step length and symmetry [36] during gait training without BWS. Variety in the applied BWS level was small between these studies and differed between 20 and 30%, suggesting that other factors such as (severity of) the disorder may explain differences between studies. Since goals were achieved in all included studies, it cannot be estimated if goal attainment was more likely to be achieved at particular BWS levels or within specific diagnoses. Variation in study designs, populations and outcome measures does not allow a to draw conclusions on the effectiveness of applied BWS levels. Therefore, further research is needed to obtain more insight into the effectiveness of specific BWS levels within particular diagnoses.

Although a considerable amount of literature exists on BWS training in rehabilitation, the variety in study characteristics preclude a clear picture of how to set BWS levels in clinical practice. Further research is necessary to develop guidelines for BWS level selection. In order to create a more comprehensive and complete overview, future studies should more clearly report, for each patient individually and for each training session within the training period: patient characteristics, the level of BWS applied and training goals pursued as well as the rationale for the applied levels. Reporting these characteristics for each individual and training session separately would allow to monitor individual progression of BWS level selection during rehabilitation. It should be noted that the training goals described in the current study were based on the reported outcome measures of the included studies. Although these are likely to be associated with training goals, future studies may investigate the relationship between BWS levels and training goals more directly, using training goals identified by therapists. Moreover, it would be useful to conduct controlled experiments to assess the effectiveness of multiple BWS levels for several diagnoses. Previous research suggested that parameter selection in robotic gait training devices might have an influence on the effectiveness of gait training [37] and argued that key-determinants for meaningful clinical use of robotic gait training devices are optimal patient selection and optimal adaptation of the device and its settings to the individual situation and goals of a patient [28]. The current study shows that variety exists in the reported BWS levels, patient characteristics and training goals. This variety indicates that BWS selection cannot be based on one general guideline, but requires multiple factors to be taken into account, such as training goals, the time point of training and patients’ level of walking ability, to develop an individually-tailored BWS training program. Therefore, future guidelines should not consist of a general advice per diagnosis, but a set of advices that can be used complementary to each other to select an appropriate BWS level for each individual.

This study has some limitations to consider for interpretation and future research. Due to the diverse and limited amount of data reported in the included studies, the influence of confounding factors such as severity of the disorder and changes within training on BWS levels and their selection could not be investigated, as well as the individual progression of BWS levels during rehabilitation. Moreover, variation in study designs and populations, and the limited amount of studies available hamper a systematic comparison of training effects. Therefore, our results do not allow conclusions to be drawn about the effect of BWS levels on rehabilitation success. In addition, studies on exoskeletons were excluded from this review, as these devices provide different types of support next to BWS, which could otherwise have influenced our results. However, as exoskeletons are also frequently used in current rehabilitation, future research may investigate in which specific circumstances BWS devices and exoskeletons should be used. These limitations should be taken into account when developing guidelines based on this review and could be topics of interest for future research.

## Conclusion

This systematic review provides a detailed overview of the initial BWS levels used during gait training in individuals with neurological impairments. We showed that BWS levels differ considerably between studies and tend to differ between diagnoses, types of BWS devices and within training goals. Our findings show that consensus on selecting BWS levels is currently lacking, as well as clarity on the underlying reasons for selecting BWS levels. Further research is necessary to reach consensus on selecting BWS levels and to experimentally investigate which levels are optimal for specific diagnoses and training goals. This review serves as a starting point for debate on selecting appropriate BWS levels in clinical practice.

### Electronic supplementary material

Below is the link to the electronic supplementary material.

Supplementary Material 1

## Data Availability

No datasets were generated or analysed during the current study.

## Abbreviations

BWS: Body weight support
CM: Ceiling-mounted
CP: Cerebral palsy
FB: Frame-based
MS: Multiple sclerosis
NOS: Newcastle-Ottawa scale
PRISMA: Reporting items for systematic reviews and meta-analyses
PROSPERO: Prospective register of systematic reviews
SCI: Spinal cord injury
TBI: Traumatic brain injury
