# Supplementary material for: Clinical indications and protocol considerations for selecting initial body weight support levels in gait rehabilitation: a systematic review
Source: J Neuroeng Rehabil. 2024 Jun 7;21:97. doi: 10.1186/s12984-024-01389-8 (PMC11157893; doi:10.1186/s12984-024-01389-8)
Supplement: Supplementary file 1 — Supplementary Material 1 [file 12984_2024_1389_MOESM1_ESM.docx]

**Appendix 1 – PRISMA guidelines**

| **Section and Topic** | **Item #** | **Checklist item** | **Location where item is reported** |
| --- | --- | --- | --- |
| **TITLE** | | |  |
| Title | 1 | Identify the report as a systematic review. | Title page |
| **ABSTRACT** | | |  |
| Abstract | 2 | See the PRISMA 2020 for Abstracts checklist. | N.A. |
| **INTRODUCTION** | | |  |
| Rationale | 3 | Describe the rationale for the review in the context of existing knowledge. | Page 3+4 |
| Objectives | 4 | Provide an explicit statement of the objective(s) or question(s) the review addresses. | Page 4 |
| **METHODS** | | |  |
| Eligibility criteria | 5 | Specify the inclusion and exclusion criteria for the review and how studies were grouped for the syntheses. | Page 5 |
| Information sources | 6 | Specify all databases, registers, websites, organisations, reference lists and other sources searched or consulted to identify studies. Specify the date when each source was last searched or consulted. | Page 5 |
| Search strategy | 7 | Present the full search strategies for all databases, registers and websites, including any filters and limits used. | Appendix 2 |
| Selection process | 8 | Specify the methods used to decide whether a study met the inclusion criteria of the review, including how many reviewers screened each record and each report retrieved, whether they worked independently, and if applicable, details of automation tools used in the process. | Page 5 |
| Data collection process | 9 | Specify the methods used to collect data from reports, including how many reviewers collected data from each report, whether they worked independently, any processes for obtaining or confirming data from study investigators, and if applicable, details of automation tools used in the process. | Page 5 |
| Data items | 10a | List and define all outcomes for which data were sought. Specify whether all results that were compatible with each outcome domain in each study were sought (e.g. for all measures, time points, analyses), and if not, the methods used to decide which results to collect. | Page 5 |
|  | 10b | List and define all other variables for which data were sought (e.g. participant and intervention characteristics, funding sources). Describe any assumptions made about any missing or unclear information. | Page 5 |
| Study risk of bias assessment | 11 | Specify the methods used to assess risk of bias in the included studies, including details of the tool(s) used, how many reviewers assessed each study and whether they worked independently, and if applicable, details of automation tools used in the process. | Page 6 |
| Effect measures | 12 | Specify for each outcome the effect measure(s) (e.g. risk ratio, mean difference) used in the synthesis or presentation of results. | N.A. |
| Synthesis methods | 13a | Describe the processes used to decide which studies were eligible for each synthesis (e.g. tabulating the study intervention characteristics and comparing against the planned groups for each synthesis (item #5)). | Page 5 |
|  | 13b | Describe any methods required to prepare the data for presentation or synthesis, such as handling of missing summary statistics, or data conversions. | N.A. |
|  | 13c | Describe any methods used to tabulate or visually display results of individual studies and syntheses. | N.A. |
|  | 13d | Describe any methods used to synthesize results and provide a rationale for the choice(s). If meta-analysis was performed, describe the model(s), method(s) to identify the presence and extent of statistical heterogeneity, and software package(s) used. | N.A. |
|  | 13e | Describe any methods used to explore possible causes of heterogeneity among study results (e.g. subgroup analysis, meta-regression). | N.A. |
|  | 13f | Describe any sensitivity analyses conducted to assess robustness of the synthesized results. | N.A. |
| Reporting bias assessment | 14 | Describe any methods used to assess risk of bias due to missing results in a synthesis (arising from reporting biases). | Page 6 |
| Certainty assessment | 15 | Describe any methods used to assess certainty (or confidence) in the body of evidence for an outcome. | N.A. |
| **RESULTS** | | |  |
| Study selection | 16a | Describe the results of the search and selection process, from the number of records identified in the search to the number of studies included in the review, ideally using a flow diagram. | Appendix 5 |
|  | 16b | Cite studies that might appear to meet the inclusion criteria, but which were excluded, and explain why they were excluded. | Page 6 |
| Study characteristics | 17 | Cite each included study and present its characteristics. | Table 1 |
| Risk of bias in studies | 18 | Present assessments of risk of bias for each included study. | Appendix 6 |
| Results of individual studies | 19 | For all outcomes, present, for each study: (a) summary statistics for each group (where appropriate) and (b) an effect estimate and its precision (e.g. confidence/credible interval), ideally using structured tables or plots. | Page 7+8+9 |
| Results of syntheses | 20a | For each synthesis, briefly summarise the characteristics and risk of bias among contributing studies. | Page 10 |
|  | 20b | Present results of all statistical syntheses conducted. If meta-analysis was done, present for each the summary estimate and its precision (e.g. confidence/credible interval) and measures of statistical heterogeneity. If comparing groups, describe the direction of the effect. | Page 7+8+9 |
|  | 20c | Present results of all investigations of possible causes of heterogeneity among study results. | Page 7+8+9 |
|  | 20d | Present results of all sensitivity analyses conducted to assess the robustness of the synthesized results. | N.A. |
| Reporting biases | 21 | Present assessments of risk of bias due to missing results (arising from reporting biases) for each synthesis assessed. | N.A. |
| Certainty of evidence | 22 | Present assessments of certainty (or confidence) in the body of evidence for each outcome assessed. | N.A. |
| **DISCUSSION** | | |  |
| Discussion | 23a | Provide a general interpretation of the results in the context of other evidence. | Page 10 |
|  | 23b | Discuss any limitations of the evidence included in the review. | Page 14 |
|  | 23c | Discuss any limitations of the review processes used. | Page 14 |
|  | 23d | Discuss implications of the results for practice, policy, and future research. | Page 14 |
| **OTHER INFORMATION** | | |  |
| Registration and protocol | 24a | Provide registration information for the review, including register name and registration number, or state that the review was not registered. | Page 4 |
|  | 24b | Indicate where the review protocol can be accessed, or state that a protocol was not prepared. | Page 4 |
|  | 24c | Describe and explain any amendments to information provided at registration or in the protocol. | N.A. |
| Support | 25 | Describe sources of financial or non-financial support for the review, and the role of the funders or sponsors in the review. | Page 16 |
| Competing interests | 26 | Declare any competing interests of review authors. | Page 16 |
| Availability of data, code and other materials | 27 | Report which of the following are publicly available and where they can be found: template data collection forms; data extracted from included studies; data used for all analyses; analytic code; any other materials used in the review. | N.A. |

*From:*  Page MJ, McKenzie JE, Bossuyt PM, Boutron I, Hoffmann TC, Mulrow CD, et al. The PRISMA 2020 statement: an updated guideline for reporting systematic reviews. BMJ 2021;372:n71. doi: 10.1136/bmj.n71

For more information, visit: <http://www.prisma-statement.org/>

**Appendix 2 – Search strategy**

*PubMed*

| **Block # 1**  **neurologic gait disorders** | "Gait Disorders, Neurologic"[Mesh] OR "Nervous System Diseases"[Mesh] OR “stroke”[tiab] OR “cerebral pals*”[tiab] OR “brain injur*”[tiab] OR “brain diseas*”[tiab] OR “cva”[tiab] OR “cerebral accident*”[tiab] OR “spinal cord injur*”[tiab] OR “multiple scleros*”[tiab] | 3,019,449 |
| --- | --- | --- |
| **Block # 2**  **BWS** | “weight support*”[tiab] OR “weight unload*”[tiab] | 1,401 |
| **Block # 3**  **locomotion** | (“locomotion”[Mesh] OR “Gait Analysis”[MeSH] OR “Walk Test”[MeSH] OR “locomot*”[tiab] OR “walk*”[tiab] OR “gait*”[tiab] OR “ambulat*”[tiab] OR “exercise*”[tiab]) | 878,226 |
| **Block # 4**  **combination of terms** | #1 AND #2 AND #3 NOT ("Animals"[Mesh] NOT "Humans"[Mesh]) | 687 |

*EMBASE*

| **Block # 1**  **neurologic gait disorders** | ‘Gait Disorders, Neurologic’/exp OR ‘Nervous System Diseases’/exp OR ‘stroke’:ti,ab OR ‘cerebral pals*’:ti,ab OR ‘brain injur*’:ti,ab OR ‘brain diseas*’:ti,ab OR ‘cva’:ti,ab OR ‘cerebral accident*’:ti,ab OR ‘spinal cord injur*’:ti,ab OR ‘multiple scleros*’:ti,ab | 4,535,039 |
| --- | --- | --- |
| **Block # 2**  **BWS** | ‘weight support*’:ti,ab OR ‘weight unload*’:ti,ab | 1,867 |
| **Block # 3**  **locomotion** | ‘locomotion’/exp OR ‘Gait Analysis’/exp OR ‘Walk Test’/exp OR ‘locomot*’:ti,ab OR ‘walk*’:ti,ab OR ‘gait*’:ti,ab OR ‘ambulat*’:ti,ab OR ‘exercise*’/ti,ab | 988,750 |
| **Block # 4**  **combination of terms** | #1 AND #2 AND #3 NOT (‘Animals’/exp NOT ‘Humans’/exp) | 992 |

*Web of Science*

| **Block # 1**  **neurologic gait disorders** | TS = (“Gait Disorders, Neurologic” OR “Nervous System Diseases” OR “stroke” OR “cerebral pals*” OR “brain injur*” OR “brain diseas*”OR “cva” OR “cerebral accident*” OR “spinal cord injur*” OR “multiple scleros*”) | 752,376 |
| --- | --- | --- |
| **Block # 2**  **BWS** | TS = ( “weight support*” OR “weight unload*”) | 2,290 |
| **Block # 3**  **locomotion** | TS = (“locomotion” OR “Gait Analysis” OR “Walk Test” OR “locomot*” OR “walk*” OR “gait*” OR “ambulat*” OR “exercise*”) | 1,009,910 |
| **Block # 4**  **combination of terms** | #1 AND #2 AND # 3 NOT TS=(“Animals” NOT “Humans”) | 1,352 |

**Appendix 3**

*Categories used to group training goals based on outcome measures*

| **Training goals** | **Keywords** |
| --- | --- |
| Improving functions of the cardiovascular system | Heart  Veins  Blood  Circulatory system |
| Improving muscle power functions | Muscle activity  Strength |
| Improving metabolic functions | Energy cost  Energy expenditure  Oxygen consumption  Metabolism  Fitness  Homeostasis |
| Improving joint mobility functions | Kinematics |
| Improving gait pattern functions | Speed  Step length  Step width  Gait symmetry  Stance and swing time or phase |
| Improving functional ability | FAC, BBS, TuG, Fugl-Meyer, etc. |
| Improving mental functions | Quality of life  Fatigue |
| Improving involuntary movement reaction functions | Balance  Upright posture |
| Improving musculoskeletal functions | Atrophy |
|  | Bone |
| Improving sensory functions and pain | Pain  Sensitivity |
| Improving voluntary movement functions | Brain  Nervous system |

**Appendix 4 – Modified Newcastle Ottawa Scale**

*Selection*

1. Representativeness of patient group (1)

- *One star was awarded when in- and exclusion criteria were described.*

1. Representativeness of patient group (2)

- *One star was awarded when patient characteristics were described (i.e. age, sex, diagnosis, time since injury).*

1. Selection of patient group

- *Studies that provided a detailed description of the recruitment of patients were awarded a star (where were patients included, how many patients were screened, and how many of them eventually participated).*

*Outcome*

1. Assessment of outcome (1)

- *One star was awarded if the applied protocol was clearly described in terms of training duration and frequency.*

1. Assessment of outcome (2)

- *One star was awarded if the applied protocol was clearly described in terms of the amount of BWS provided during training.*

1. Follow-up adequacy

- *One star was awarded if ≤ 10% of the subjects that were initially included dropped out of the study / were not included in the final analysis.*

**Appendix 5 – Flow of search strategy**


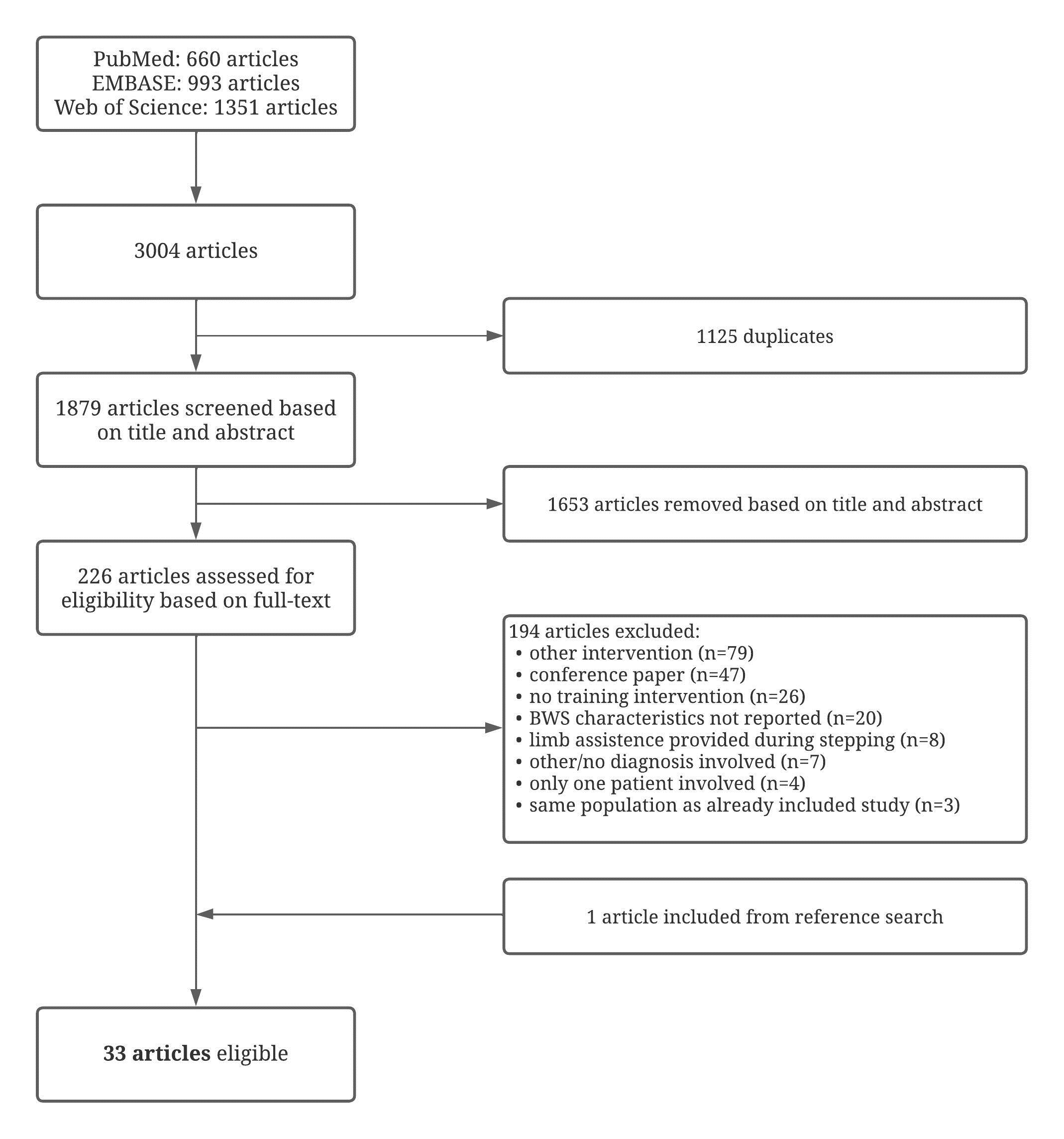


**Appendix 6 – Scoring Newcastle Ottawa Scale**

| **Study** | **Item 1** | **Item 2** | **Item 3** | **Item 4** | **Item 5** | **Item 6** | **NOS**  **score** |
| --- | --- | --- | --- | --- | --- | --- | --- |
| Abel et al., 2002 | **🟑** |  |  | **🟑** | **🟑** | **?** | **3** |
| Alexeeva et al., 2011 | **🟑** | **🟑** | **🟑** | **🟑** | **🟑** | **🟑** | **6** |
| Chua et al., 2020 | **🟑** | **🟑** | **🟑** | **🟑** | **🟑** | **🟑** | **6** |
| Combs et al., 2013 | **🟑** | **🟑** |  | **🟑** | **🟑** |  | **4** |
| Combs et al., 2014 | **🟑** | **🟑** | **🟑** | **🟑** | **🟑** | **🟑** | **6** |
| Effing et al., 2006 |  | **🟑** |  | **🟑** | **🟑** | **?** | **3** |
| Esquenazi et al., 2013 | **🟑** |  | **🟑** | **🟑** |  | **🟑** | **5** |
| Esquenazi et al., 2017 | **🟑** | **🟑** |  | **🟑** | **🟑** | **🟑** | **5** |
| Gama et al., 2017 | **🟑** | **🟑** | **🟑** | **🟑** | **🟑** |  | **5** |
| Gazzani et al., 1999 |  | **🟑** |  | **🟑** | **🟑** | **?** | **3** |
| Geroin et al., 2011 | **🟑** | **🟑** | **🟑** | **🟑** |  | **🟑** | **5** |
| Giangregorio et al., 2006 | **🟑** | **🟑** | **🟑** | **🟑** | **🟑** | **🟑** | **6** |
| Gorassini et al., 2009 | **🟑** | **🟑** |  | **🟑** | **🟑** |  | **4** |
| Graham et al., 2018 | **🟑** | **🟑** | **🟑** | **🟑** | **🟑** |  | **5** |
| Hicks et al., 2005 | **🟑** | **🟑** |  | **🟑** | **🟑** |  | **4** |
| Ivanenko et al., 2003 |  |  |  | **🟑** | **🟑** |  | **2** |
| Kim et al., 2020 | **🟑** | **🟑** | **🟑** | **🟑** | **🟑** | **🟑** | **6** |
| Kim et al., 2014 | **🟑** | **🟑** |  | **🟑** |  | **?** | **3** |
| Martin Ginis et al., 2007 | **🟑** | **🟑** |  | **🟑** | **🟑** |  | **4** |
| Miller et al., 2002 |  | **🟑** |  | **🟑** | **🟑** | **?** | **3** |
| Moore et al., 2010 | **🟑** | **🟑** |  |  | **🟑** |  | **3** |
| Musselman et al., 2009 |  | **🟑** |  | **🟑** |  |  | **2** |
| Philips et al., 2004 |  |  |  | **🟑** | **🟑** | **?** | **2** |
| Piira et al., 2020 | **🟑** | **🟑** |  | **🟑** | **🟑** |  | **4** |
| Pilutti et al., 2011 | **🟑** | **🟑** |  | **🟑** | **🟑** | **🟑** | **5** |
| Pilutti et al. , 2016 | **🟑** | **🟑** |  | **🟑** | **🟑** |  | **4** |
| Protas et al. , 2001 | **🟑** | **🟑** |  | **🟑** | **🟑** | **?** | **4** |
| Ribeiro et al., 2013 | **🟑** |  |  | **🟑** | **🟑** | **?** | **3** |
| Sousa et al. , 2011 | **🟑** | **🟑** | **🟑** | **🟑** | **🟑** |  | **5** |
| Stewart et al., 2004 | **🟑** | **🟑** |  | **🟑** | **🟑** | **?** | **4** |
| Sullivan et al., 2002 | **🟑** | **🟑** |  | **🟑** | **🟑** | **?** | **4** |
| Takao et al., 2015 | **🟑** | **🟑** |  | **🟑** | **🟑** | **🟑** | **5** |
| Trueblood, 2001 | **🟑** |  |  | **🟑** | **🟑** | **?** | **3** |
